# Decreased Phase–Amplitude Coupling Between the mPFC and BLA During Exploratory Behaviour in Chronic Unpredictable Mild Stress-Induced Depression Model of Rats

**DOI:** 10.3389/fnbeh.2021.799556

**Published:** 2021-12-16

**Authors:** Zihe Wang, Qingying Cao, Wenwen Bai, Xuyuan Zheng, Tiaotiao Liu

**Affiliations:** School of Biomedical Engineering and Technology, Tianjin Medical University, Tianjin, China

**Keywords:** depression, medial prefrontal cortex (mPFC), basolateral amygdala (BLA), phase–amplitude coupling (PAC), theta–gamma coupling, exploratory behaviour

## Abstract

Depression is a common neuropsychiatric illness observed worldwide, and reduced interest in exploration is one of its symptoms. The control of dysregulated medial prefrontal cortex (mPFC) over the basolateral amygdala (BLA) is related to depression. However, the oscillation interaction in the mPFC-BLA circuit has remained elusive. Therefore, this study used phase–amplitude coupling (PAC), which provides complicated forms of information transmission by the phase of low-frequency rhythm, modulating the amplitude of high-frequency rhythm, and has a potential application for the treatment of neurological disease. The chronic unpredictable mild stress (CUMS) was used to prepare the rat models of depression. Moreover, multichannel *in vivo* recording was applied to obtain the local field potentials (LFPs) of the mPFC, the BLA in rats in control, and CUMS groups, while they explored the open field. The results showed prominent coupling between the phase of theta oscillation (4–12 Hz) in the mPFC and the amplitude of high-gamma oscillation (70–120 Hz) in the BLA. Compared to the control group, this theta–gamma PAC was significantly decreased in the CUMS group, which was accompanied by the diminished exploratory behaviour. The results indicate that the coupling between the phase of theta in the mPFC and the amplitude of gamma in the BLA is involved in exploratory behaviour, and this decreased coupling may inhibit exploratory behaviour of rats exposed to CUMS.

## Introduction

As one of the major leading causes of burden in diseases, depression is a common neuropsychiatric illness affecting over 320 million people globally (World Health Organization, 2017). There are several symptoms shown in depression, loss of pleasure, feeling of hopelessness, lack of motivation, reduced interest in exploration, and even suicidal thoughts (Zhou et al., 2021).

Depression, for which stress is a main risk factor, is related to dysregulated medial prefrontal cortex (mPFC) control over the basolateral amygdala (BLA). The mPFC is commonly involved in behavioural regulation and autonomic responses to stress (Czéh et al., 2007; Dias-Ferreira et al., 2009). Structural changes occur in the mPFC, including loss of spines and retraction of dendrites, after an individual is exposed to stress (Goldwater et al., 2009; Gilabert-Juan et al., 2013). The BLA is involved in the expression of negative emotions such as fear, depression, and anxiety (Tye et al., 2011; Dias et al., 2013; Leuner and Shors, 2013). Repeated stress can enhance the dendritic length and the quantity of spine in the BLA, which is consistent with the increase in the firing rate and membrane excitability in BLA neurons (Leuner and Shors, 2013; Padival et al., 2015). The mPFC and BLA are widely interconnected and, together, participated in the regulation of depression. In general, the mPFC inhibits the activity of the BLA, limiting the output of the BLA to prevent the expression of negative emotions (Shansky and Morrison, 2009; Motzkin et al., 2015; Hultman et al., 2016). Meanwhile, PFC inputs to the BLA selectively drive feedback projections to the PFC (McGarry and Carter, 2017). Another study from the aspect of the amplitude of neural oscillations, using directed transfer function, found that the connection of the mPFC network in rats exposed to chronic unpredictable mild stress (CUMS) was decreased, which reduced information transmission from the mPFC to the BLA during exploratory behaviour (Qi et al., 2020). The mPFC and BLA are also related to exploratory behaviour, which is one of the most basic activities of animals and is reduced in chronically stressed rats (Jacinto et al., 2013; Liu et al., 2020). These pieces of research indicate that the interaction between the mPFC and BLA is engaged in the regulation of negative emotions through inhibiting the activity of the BLA, and a decreased information communication between the mPFC and BLA may lead to depression-like behaviours.

There are neural oscillations of different rhythms, which can provide complicated forms of information transmission through interaction when the brain receives, integrates, and processes information, including delta oscillations (1–4 Hz), theta oscillations (4–12 Hz), beta oscillations (12–30 Hz), low-gamma oscillations (30–70 Hz), and high-gamma oscillations (70–120 Hz). Phase–amplitude coupling (PAC) represents the coupling between the phase of a low-frequency oscillation and the amplitude/power of a high-frequency oscillation (Seymour et al., 2017). Prior pieces of research suggest that PAC may facilitate the communication of neuronal information at different time and space scales, which potentially provides a mechanism for synchronisation and communication between local and global processes in the brain (van der Meij et al., 2012; Florin and Baillet, 2015). Moreover, changes in PAC are closely associated with neurological disorders (Noda et al., 2017). Given the inseparable relationship between the mPFC-BLA and depression as well as the essential role of the interaction between neural oscillations in depression, it is important to detect the PAC between the phase of mPFC and amplitude of BLA in rats exposed to CUMS for better understanding of the information interaction within these two brain regions.

To address this problem, CUMS was used for preparation of the rat model of depression. Multichannel *in vivo* recording was applied to obtain the local field potentials (LFPs) of the mPFC, BLA in the control, and the CUMS groups of rats during exploration in an open field. LFPs are widely used to reflect synaptic potentials from a large number of neurons because of their high spatiotemporal resolution (Marceglia et al., 2007). The open field test (OFT) is a common method that can quantitatively detect exploratory behaviour (Belovicova et al., 2017). The modulation index (MI) was calculated to quantitatively analyse the PAC of the mPFC-BLA circuit of rats in the open field to probe the neuronal information communication mechanism during the exploratory behaviour (Tort et al., 2010).

## Materials and Methods

All procedures mentioned here conformed to the Guide for the Care and Use of Laboratory Animals and approved by the Animal Care and Use Committee of Tianjin Medical University.

### Animals

Experimental animals were male Sprague–Dawley rats (10–12 weeks, 300–350 g) obtained from the Experimental Animal Centre of Tianjin Medical University in China. The animals were divided randomly into the control and CUMS groups. These rats were kept (three to four per cage) in a room (25 ± 2^°^C) with the humidity maintained at 45–55%. Unless otherwise specified, the rats were housed under a 12-h light–dark cycle and allowed to feed and drink freely.

### Preparation and Verification of the CUMS-Induced Depression Model in Rats

The CUMS has been found to result in increased behavioural despair and the inability to experience pleasure (anhedonia) in rodents, which is one of the central symptoms in depression (Muscat et al., 1992; Willner, 1997), so that it is recognised as the most widespread, reliable, and effective method for depression model in rodent (Antoniuk et al., 2019). In this study, the rats in the CUMS group were subjected to six kinds of repeated stressors in random order for 21 days, including a tail pinch (1 min), ice water swimming (4^°^C, 5 min), food and water deprivation (24 h), placement into a tilted cage (45° tilt, 12 h), a reversed light/dark cycle (24 h), and wet padding (12 h).

The sucrose preference test (SPT) and forced-swim test (FST) were applied to evaluate the depression model of rats in depression after CUMS. For the SPT, the rats were first trained to consume 1% sucrose from two bottles for 24 h to acclimate them to sucrose, and then the animals were allowed free access to 1% sucrose and water from two bottles for 12 h. After 24-h deprivation of food and water, the animals were provided with 1% sucrose and water in two bottles for 1 h. The preference rate for sucrose (the sucrose preference rate = sucrose consumption/total fluid consumption × 100%) was recorded.

For the FST, the animals were placed in a transparent cylinder bath (30 cm in diameter, 100 cm in height) filled of 25°C (± 2°C) water for 5 min after the pretest (15 min). The interval between pretest and test is 24 h. The full 5-min immobility time was scored (immobility was defined as lack of activity, except if it is necessary to keep the head above water). The preference rate for sucrose in SPT and the immobility time in the FST were recorded and compared between the two groups.

### Surgery

**FIGURE 1:**
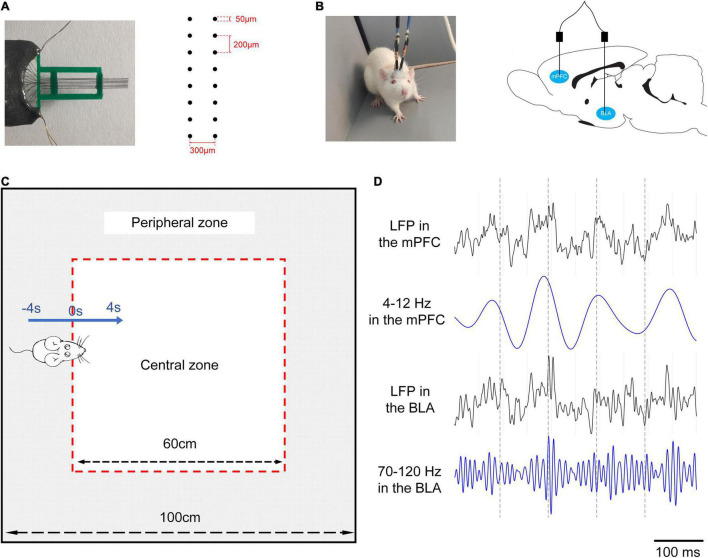
An experimental scheme. **(A)** Left: a 16-channel microelectrode array. Right: the arrangement of the electrode array. **(B)** Left: the rat with two electrode arrays in the mPFC and BLA. Right: the position of two electrode arrays in the brains of rats. **(C)** Exploratory behaviour in the OFT. The open field (the whole area surrounded black lines, 100 cm × 100 cm) is divided into a central zone (the white area, 60 cm × 60 cm) and a peripheral zone (the grey area). The blue arrow refers to the rat going into the central zone (0–4 s) from the peripheral zone (–4 to 0 s), 0 s: Reference point (RP) is the moment when all paws of a rat entering into the central zone. Two black arrows are used to describe sizes of areas. All marks are prepared for the video analysis, and there are no marks in the behavioural test. **(D)** LFPs data during exploratory behaviour, from top to bottom: LFP in the mPFC, theta oscillations in the mPFC, LFP in the BLA, high-gamma oscillations in the BLA.

The surgery procedures were performed after successful preparation of the two groups of rats (Bai et al., 2012). Briefly, each animal was anaesthetised with pentobarbital sodium (40 mg/kg, i. p.). The coordinates of the mPFC (2.5–4.5 mm anterior to bregma, 0.2–1 mm lateral to midline, and 2.5–3 mm depth) and BLA (1.56–3.36 mm posterior to bregma, 4.4–5.2 mm lateral to midline, and 8.6–9.1 mm depth) were confirmed according to the rat brain atlas in stereotaxic coordinates. Two multichannel microelectrode arrays (2 × 8 configuration, nickel-chromium wires, < 1MΩ) were made and implanted into these two brain regions (Figures 1A,B). The electrodes were finally fixed on the skull using dental cement. Postoperatively, iodophor was given on the incision site to prevent infection. After a week of recovery, the electrophysiological signals were recorded during the OFT.

### Open Field Test

The OFT is a common measure of movement activity for rodents based on their curiosity-driven movements and thigmotaxis (spent more time along the wall). Many open-field-dependent parameters are analysed with great value for reporting, including total movement distance, time spent in the centre, vertical activity, and changes in activity over time (Gould et al., 2009). The open field in this study was a square enclosure (100 cm × 100 cm) with a surrounding wall that prevented escape. We demarcated a square area (60 cm × 60 cm) in the centre of the open field during the video analysis, which was defined as the “central zone,” while the “peripheral zone” was the other area (Figure 1C). Each animal was placed in the centre of the open field under normal illumination (185 lux) at the start, and the video-tracking system (Sony, Japan) was turned on to record the activities of the animals for 10 min. A trial of exploratory behaviour was defined as the movement of the rats going into the central zone from the peripheral zone, including 4 s in the peripheral zone and 4 s in the central zone. The data that the rat spent less than 4 s in the central zone were excluded to ensure the availability of recordings. After every test, 75% alcohol was used to clean the open field thoroughly for removing odours. The position of the animals was detected by the Ethovision XT 8.5 software (Noldus Information Technology, Netherlands), and the behavioural parameters were calculated, including the total distance moved, the rearing times, the entries of the central zone, and the time spent in the central zone.

### Data Acquisition and Preprocessing

The electrophysiological signals were recorded by Neural Data Acquisition System (Plexon, United States), and the position of the rats was obtained simultaneously by the overhead camera. The raw data underwent a series of processing, including amplification (gain: 5,000), bandpass filtering (0.3–120 Hz), and sampling at 2 kHz to obtain LFPs. Then, 50-Hz notch filter and polynomial fitting were applied to remove the powerline artefact and the baseline drifts. At last, the delta, theta, beta, low-gamma, and high-gamma bands were extracted by a bandpass filter (Figure 1D shows an example that theta oscillations in the mPFC and gamma oscillations in the BLA can be, respectively, extracted from LFPs).

### Phase–Amplitude Coupling Analysis

Modulation index was commonly used to measure the strength of PAC (Tort et al., 2010), which was mainly based on the non-uniform distribution of the high-frequency amplitude on the low-frequency phase signal. The Shannon entropy was calculated to characterise the non-uniformity of distribution, and the maximum value of Shannon entropy under a uniform distribution was used for normalisation.

First, the time series of the phases [denoted as ϕ*f*_*p*_(*t*)] and the amplitude envelope [denoted as *Af*_*A*_(*t*)] was extracted by Hilbert, transformed from the filtered LFPs at two frequency ranges under analysis. Note that the bandwidth for the phase component must be narrow, while the bandwidth for the high-frequency component must include the side peaks generated by the low-frequency rhythm (Berman et al., 2012; Aru et al., 2015; Seymour et al., 2017). Then, the composite time series [ϕ*f*_*p*_(*t*), *Af*_*A*_(*t*)] was produced, which included the corresponding amplitude series at each phase series. The phase series were divided into 18 bins (each included 20°), and the mean amplitude over each phase bin was calculated. The mean amplitude *Af*_*A*_(*t*) at the phase bin *i* was denoted by < *Af*_*A*_ > ϕ*f*_*p*_(*i*), and it was normalised through dividing each bin value by the sum over the bins:

(1) $$P\,\left(i\right)=\frac{{<A_{f_{A}}>}_{\phi_{f_{p}}\,\left(i\right)}}{\sum_{k=1}^{N}{<A_{f_{A}}>}_{\phi_{f_{p}}\,\left(k\right)}}$$

where *N* is the number of phase bins (*N* = 18 in this article). Then, Shannon entropy based on the *P*(*i*) above was given by:

(2) $$H\,\left(P\right)=-\sum_{i=1}^{N}P\,\left(i\right)\cdot l\,o\,g\,\left[P\,\left(i\right)\right]$$

Notice that *log*(*N*) was the maximal value of Shannon entropy, which precisely occurred for the uniform distribution [(*P*(*i*) = 1/*N* for all bins *i*)]. The MI was defined as normalised Shannon entropy:

(3) $$\mathrm{MI}=\frac{l\,o\,g\,\left(N\right)-H\,\left(P\right)}{l\,o\,g\,\left(N\right)},\mathrm{MI}\in \left[0,1\right]$$

Obviously, the greater the MI value, the stronger the PAC strength. The MI value of 0 occurs if *P*(*i*) is a uniform distribution, while the value is 1 when *P*(*i*) is a Dirac-like distribution.

### Histology

At the completion of all the experiment, the animals that deeply anaesthetised were perfused transcardially with phosphate-buffered saline (PBS) and then followed by 4% paraformaldehyde. After the dissection and storage (30% sucrose in PBS, 4^°^C) of the brain, the tissue was sectioned at 150 μm on a vibratome (Vibratome, United States). The images showing the lesion sites were obtained by an optical microscope (Olympus, Japan) and were verified histologically according to the rat brain atlas.

### Statistical Analysis

In this study, the behavioural data and PAC strength in the rats in different groups were compared. Specifically, the verification of the CUMS-induced depression model in the rats was analysed using non-paired sample *t*-test, and the comparisons of behavioural data in the OFT were evaluated by independent sample *t*-test. The electrophysiological analyses were done by two-way ANOVA, two-way repeated-measures ANOVA, and Bonferroni’s test for *post hoc* analyses. Besides, the correlation between the moving speed and MI of each subject was calculated by Pearson correlation. The distribution of electrophysiology trials in the control and CUMS groups per rat is shown in Supplementary Table 1. The statistical tests used and the results are presented in the figure legends, and the errors are reported as the mean ± SEM. *P*-values are marked statistically significant as follows: ^**^p < 0.01 and ^***^p < 0.001.

## Results

### Verification of Depression Model in Rats

**FIGURE 2:**
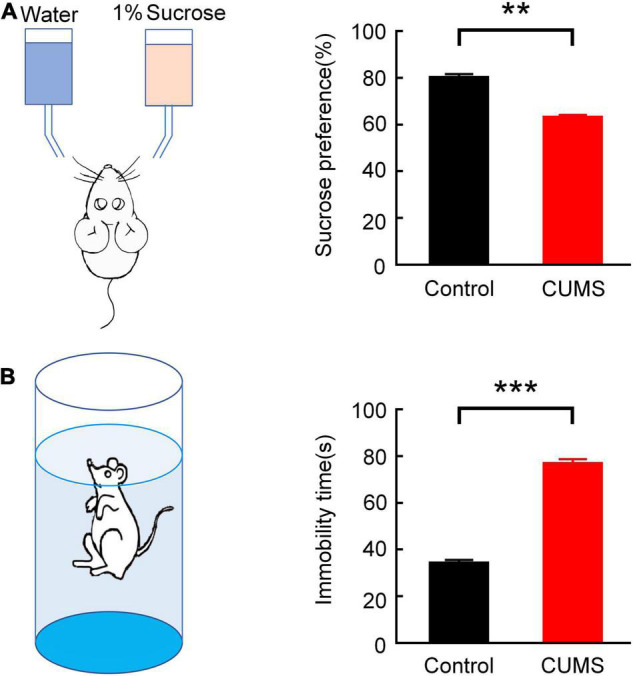
Validation of the depression model in rats. **(A)** Comparison of the sucrose preference rate between the control and CUMS groups in SPT (independent sample *t*-test, **p < 0.01). **(B)** Comparison of suspended immobility time between the control and CUMS groups in FST (independent sample *t*-test, ***p < 0.001). Error bars indicate SEM.

The statistics showed that the sucrose preference rate substantially declined (the control group: 80.07 ± 1.54%, the CUMS group: 63.10 ± 1.04%, *n* = 10 rats, *t* = 9.12, *p* < 0.01; Figure 2A), while the suspended immobility time of forced swimming was significantly prolonged (the control group: 34.110 ± 1.405 s, the CUMS group: 76.810 ± 1.934 s, *n* = 10 rats, *t* = 16.16, *p* < 0.001; Figure 2B). The results demonstrated that there was a prominent difference in the behavioural despair (FST) and the pleasure response to reward (SPT) after CUMS procedure, which suggested that the CUMS-induced depression rats were prepared successfully.

### Behavioural Performance

**FIGURE 3:**
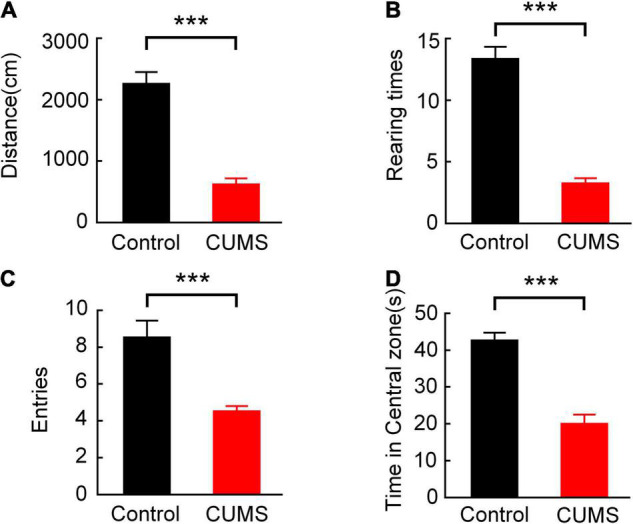
Comparison of behavioural performance between the control and CUMS groups in OFT. **(A)** Comparison of the total distance moved (independent sample *t*-test, ***p < 0.001). **(B)** Comparison of the rearing times (independent sample *t*-test, ***p < 0.001). **(C)** Comparison of the entries of the central zone (independent sample *t*-test, ***p < 0.001). **(D)** Comparison of the time spent in the central zone (independent sample *t*-test, ***p < 0.001). Error bars indicate SEM.

After the verification of the CUMS group, the behavioural parameters of the two-group animals were recorded during the 10-min test in the open field. Figure 3 shows that the total distance moved (the control group: 2251.989 ± 198.699 cm, the CUMS group: 614.542 ± 103.570 cm, *n* = 10 rats, *t* = 7.31, *p* < 0.001, Figure 3A), the rearing times (the control group: 13.300 ± 1.033, the CUMS group: 3.200 ± 0.467, *n* = 10 rats, *t* = 8.91, *p* < 0.001, Figure 3B), the entries of the central zone (the control group: 8.500 ± 0.946, the CUMS group: 4.500 ± 0.307, *n* = 10 rats, *t* = 4.02, *p* < 0.001, Figure 3C), and the time spent in the central zone (the control group: 42.570 ± 2.201 s, the CUMS group: 19.883 ± 2.632 s, *n* = 10 rats, *t* = 6.61, *p* < 0.001, Figure 3D) in the CUMS group were all significantly lower than those in the control group. These results suggested that exploratory behaviour of the rats exposed to CUMS was diminished.

### Histology

**FIGURE 4:**
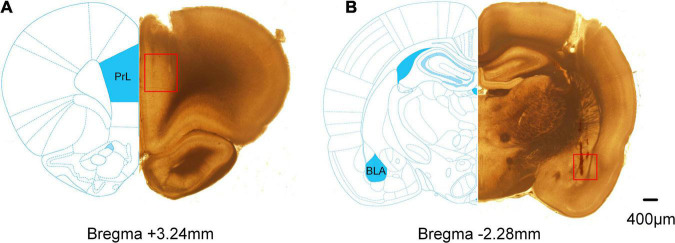
Histological examinations of the electrode sites in the mPFC and BLA. Compared partial brain sections with rat brain atlas. The red squares mark the position of the electrode tips. **(A)** The lesion sites in the mPFC. **(B)** The lesion sites in the BLA.

Extracellular electrophysiological recordings were obtained from the mPFC and BLA in rats. The representative histological examinations of lesion sites in the mPFC and BLA are shown in Figure 4.

### Decreased Theta–Gamma PAC From mPFC to BLA in Rats Exposed to CUMS

**FIGURE 5:**
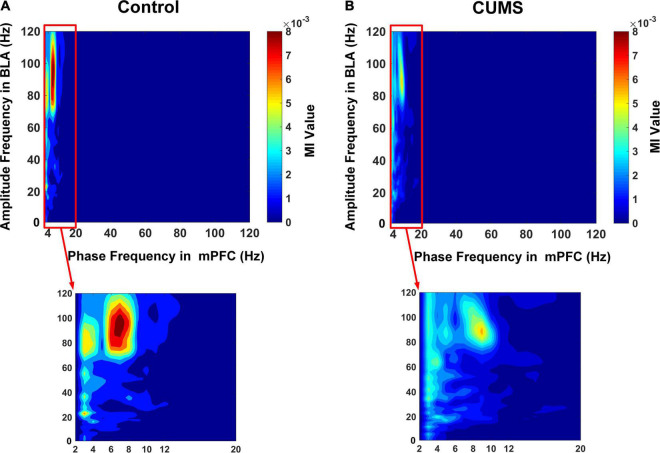
Phase–amplitude comodulograms between the mPFC and BLA. **(A)** The mPFC phase–BLA amplitude coupling in the control group. **(B)** The mPFC phase–BLA amplitude coupling in the CUMS group. Warm colours show stronger coupling; note the prominent coupling between the theta phase in the mPFC and high-gamma amplitude in the BLA.

The LFPs in mPFC and BLA were analysed, and the MI was calculated in two groups at different frequencies. The comodulogram at all bands showed there was a prominent modulation between the theta phase in the mPFC and a high-gamma amplitude in the BLA (Figure 5A: mPFC phase–BLA amplitude coupling in the control group, *n* = 10 rats, 200 trials). The characteristics of mPFC phase–BLA amplitude coupling in the CUMS group were similar to those in the control group (Figure 5B: mPFC phase–BLA amplitude coupling in the CUMS group, *n* = 10 rats, 200 trials). This finding indicated that the high-gamma amplitude in the BLA was strongly coupled with the theta phase in the mPFC and that mPFC theta-BLA high-gamma PAC was involved in the exploratory behaviour, so we mainly focused on the theta–gamma PAC for further analysis.

**FIGURE 6:**
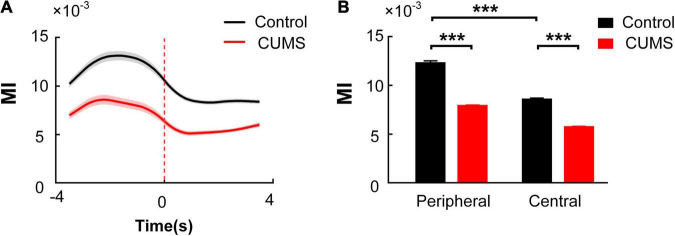
Comparison of theta-high-gamma PAC in the mPFC and BLA during exploratory behaviour between the control and CUMS groups. **(A)** Dynamic changes of MI in the control (black) and CUMS (red) groups. The red dotted line indicates the RP, and the shaded areas represent ± SEM. **(B)** Multiple comparisons of the averaged MI in the peripheral zone and the central zone in the two groups (Bonferroni’s test, ***p < 0.001). Error bars indicate SEM.

Then, we compared the MI of the mPFC theta-BLA high-gamma coupling in the rats exposed to CUMS with that in the control group. Both curves of MI value over time in the two groups were calculated and were plotted by recorded data during the 8-s exploratory behaviour period. The MI of the mPFC theta-BLA high-gamma coupling increased to a peak before the RP and then decreased gradually [two-way repeated-measures ANOVA, factors: Con/CUMS group (G) and time in the OFT (T), *n* = 10 rats, 200 trials, main effect: G:*F*_(1_,_398)_ = 362, *p* < 0.001; T: *F*_(7_,_2_,_786)_ = 65.58, *p* < 0.001; interaction: *F*_(7_,_2_,_786)_ = 5.958, *p* < 0.001. Figure 6A]. The average MI between the mPFC theta and BLA high-gamma in the CUMS group was significantly lower than that in the control group, both in the peripheral zone and in the central zone. In addition, the MI between the mPFC theta and BLA high gamma in the peripheral zone is higher than that in the central zone in the control group [two-way ANOVA, factors: Con/CUMS group (G) and the zone in the OFT (Z), *n* = 10 rats, 200 trials, main effect: G:*F*_(1_,_398)_ = 362, *p* < 0.001; Z: *F*_(1_,_398)_ = 553.4, *p* < 0.001; interaction: *F*_(1_,_398)_ = 38.8, *p* < 0.001. *Post hoc* analyses (Bonferroni’s test): The control group in the peripheral zone: 12.276 × 10^–3^ ± 0.253 × 10^–3^, the control group in the central zone: 8.561 × 10^–3^ ± 0.158 × 10^–3^, the CUMS group in the peripheral zone: 7.861 × 10^–3^ ± 0.088 × 10^–3^, the CUMS group in the central zone: 5.732 × 10^–3^ ± 0.063 × 10^–3^, the control group: periphery vs. centre, *t* = 21.04, *p* < 0.001; the periphery zone: control vs. CUMS: *t* = 19.3, *p* < 0.001; the central zone: control vs. CUMS, *t* = 12.41, *p* < 0.001; Figure 6B]. Those results revealed that the decreased mPFC theta-BLA high-gamma coupling may result in diminished exploratory behaviour in the rats exposed to CUMS.

### Correlation Between Moving Speed and MI

**FIGURE 7:**
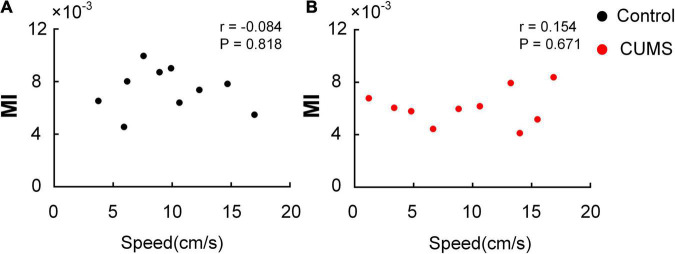
Correlation between moving speed and MI. No significant correlation is found between moving speed and MI in the open field. **(A)** The control group (*r* = –0.084, *P* = 0.818). **(B)** The CUMS group (*r* = 0.154, *P* = 0.671).

To confirm whether the moving speed of the rats affected MI, the correlation between the moving speed and MI of each subject was detected by Pearson correlation coefficient. The moving speed of the animals had no significant association with the dynamic variation of MI in the control (*r* = –0.084, *P* = 0.818, Figure 7A) and CUMS groups (*r* = 0.154, *P* = 0.671, Figure 7B). The results suggested that the task-induced increase in MI could not be correlated by the changes of moving speed of the animals.

## Discussion

This study investigated the interaction of oscillations between the mPFC and BLA during exploratory behaviour in the rats exposed to CUMS. The interaction between the mPFC and BLA was mainly based on the coupling between the phase of theta oscillation in the mPFC and the amplitude of high-gamma oscillation in the BLA. Moreover, the theta-high-gamma PAC between the mPFC and BLA was decreased along with the diminished exploratory behaviour in the rats exposed to CUMS, suggesting the decreased functional coupling between the mPFC and BLA in the rats exposed to CUMS, which may aid in monitoring the depressed state and in providing stimulation based on a specific phase to relieve depression symptoms.

The mPFC-BLA circuit is widely accepted as a key circuit to suppress negative emotions, such as depression, anxiety, and fear, *via* the projections from mPFC to the BLA (Rosenkranz and Grace, 2001; Milad and Quirk, 2002; Hare et al., 2019; Liu et al., 2020). Anatomically, there have the most axon terminals of mPFC synapsing on dendritic spines of BLA neurons, whereas only a handful of those would be on putative interneurons, so the projections from mPFC to BLA may be excitatory (Brinley-Reed et al., 1995). Given that inhibiting the BLA through the GABAergic transmission to prevent the expression of negative emotions, the projections from mPFC may activate a subset of local inhibitory network to yield feed-forward inhibition into BLA (Rosenkranz and Grace, 2001; Woodruff and Sah, 2007; Truitt et al., 2009; Liu et al., 2020). The mPFC inhibits neural activity of amygdala through the GABAergic intercalated neurons in the amygdala (Quirk et al., 2003; Amano et al., 2010). Moreover, the unpredictable stress could induce the less glutamatergic projection from the pyramidal neurons in the PFC to GABAergic interneurons in BLA, resulting in the feed-forward disinhibition of principal neurons in the BLA, which led to a variety of abnormalities in behaviour (Wei et al., 2018). This study found the decreased functional coupling between mPFC and BLA, which may represent the diminished projection from mPFC to BLA.

In the brain, the synchronised, rhythmic variations of the membrane polarisation in neurons formed oscillations. Specifically, the high-frequency oscillations reflect faster information processing in local regions of the brain, while low-frequency oscillations can project information across different brain regions based on external sensory input and internal cognitive events (Lakatos et al., 2008; Saleh et al., 2010; Salimpour and Anderson, 2019). PAC is one of the most common forms of interaction in different rhythmic oscillations. According to previous research, the activities of neuronal population are modulated in specific temporal patterns that are described by the phase of theta oscillations, and induce neural spiking and faster postsynaptic activities, which can be illustrated as gamma oscillations and broadband bursts (Haider et al., 2006; Lisman and Buzsáki, 2008; Canolty and Knight, 2010; Salimpour and Anderson, 2019). Additionally, some studies have reported that the generation of gamma oscillation relied exclusively on GABAergic interneuron in the *ex vivo* BLA (Antonoudiou et al., 2021), and the changes in gamma oscillation are associated with alterations in dendritic plasticity, especially somatostatin-positive GABAergic interneurons (Fitzgerald and Watson, 2018). In short, PAC is a good method to reflect changes in neural oscillation patterns, so that it is believed to be closely associated with various neurological diseases. In the present study, the decreased theta-high-gamma PAC was found between mPFC and BLA in the rats exposed to CUMS. Considering the inseparable relationship between PAC and depression, the theta–gamma PAC between mPFC and BLA may aid in monitoring the depressed state.

Growing evidence has suggested that it is important to synchronise the stimulation frequencies and phases with the target brain oscillations to improve the effect of stimulation (Brittain et al., 2013; Zrenner et al., 2018; Chung et al., 2019). A recent study has shown that intermittent theta-burst stimulation (iTBS), a newer form of repeated transcranial magnetic stimulation (rTMS), can make the efficiency for daily treatment of patients with depression increase several times compared with 10 Hz rTMS without compromising clinical effectiveness (Blumberger et al., 2018). Another clinical report shows that the individualised iTBS resulted in significant changes in neurophysiology and mood compared to the standard paradigms; the individualised frequency of stimulation was determined by the PAC between frontal theta (phase) and parietal gamma (amplitude) during tasks (Chung et al., 2019). Although the brain regions are different, these findings suggest that it is feasible to stimulate the target brain regions according to PAC. Therefore, based on the finding of the decreased theta-high-gamma PAC between mPFC and BLA of the rats exposed to CUMS in this study, the stimulation based on the theta phase on prefrontal cortex for relieving depression needs to be explored for further investigation.

In addition to the mPFC and BLA, neurorhythmic coupling exists in multiple brain areas, which are also associated with depression. Specifically, the hippocampus (HPC) is considered to play an important role in depression. The fMRI shows the lower functional connectivity of the HPC in patients who are depressed compared with normal controls (Song et al., 2016). The unidirectional phase coupling of theta frequency from ventral HPC (vHPC) to mPFC in the normal rats is substantially enhanced after long-term potentiation (LTP) induction, while this coupling is less enhanced in the CUMS rats (Zheng and Zhang, 2015). Likewise, the thalamus is thought as a complex emotion control node. There are significant volume reductions in the left thalamus of patients who are depressed (Zhang et al., 2018). The theta–gamma phase locking at the 1:6 ratio between the thalamus and mPFC is reduced in the rats exposed to stress (Zheng and Zhang, 2013). Overall, the formation and regulation of emotions require the participation of multiple brain regions, so more brain areas and circuits have become potential targets for the investigation of emotions.

Taken together, this study investigated the coupling between the phase of theta oscillation in the mPFC and the amplitude of gamma oscillation in the BLA, which is necessary for exploratory behaviour. Moreover, we found that the decreased coupling between the phase of the mPFC and the amplitude of BLA in the rats exposed to CUMS. These results may provide a potential mechanism for the CUMS-induced depression model of rats and contribute to future studies of the stimulation on the target brain at a specific rhythm phase to relieve depressive symptoms.

## Data Availability Statement

The raw data supporting the conclusions of this article will be made available by the authors, without undue reservation.

## Ethics Statement

The animal study was reviewed and approved by the Animal Care and Use Committee of Tianjin Medical University.

## Author Contributions

TL and XZ designed the experiments. ZW, QC, and WB carried out the experiments and analysed the data. ZW, XZ, and TL drafted the manuscript. All the authors read and approved the final version.

## Conflict of Interest

The authors declare that the research was conducted in the absence of any commercial or financial relationships that could be construed as a potential conflict of interest.

## Publisher’s Note

All claims expressed in this article are solely those of the authors and do not necessarily represent those of their affiliated organizations, or those of the publisher, the editors and the reviewers. Any product that may be evaluated in this article, or claim that may be made by its manufacturer, is not guaranteed or endorsed by the publisher.
