# Supplementary material for: Decreased Phase–Amplitude Coupling Between the mPFC and BLA During Exploratory Behaviour in Chronic Unpredictable Mild Stress-Induced Depression Model of Rats
Source: Front Behav Neurosci. 2021 Dec 16;15:799556. doi: 10.3389/fnbeh.2021.799556 (PMC8716490; doi:10.3389/fnbeh.2021.799556)
Supplement: Supplementary file 1 [file Table_1.DOCX]

Table 1

Distribution of trials in control and CUMS group for per rat.

| Rats in control group | Trials | Rats in CUMS group | Trials |
| --- | --- | --- | --- |
| 1 | 21 | 1 | 19 |
| 2 | 20 | 2 | 21 |
| 3 | 22 | 3 | 20 |
| 4 | 20 | 4 | 20 |
| 5 | 19 | 5 | 18 |
| 6  7  8  9  10 | 20  20  19  20  19 | 6  7  8  9  10 | 20  21  21  20  20 |
| Sum | 200 | Sum | 200 |
